# Labeling of mesenchymal stromal cells with iron oxide-poly(l-lactide) nanoparticles for magnetic resonance imaging: uptake, persistence, effects on cellular function and magnetic resonance imaging properties

**DOI:** 10.3109/14653249.2011.571246

**Published:** 2011-04-15

**Authors:** Gerlinde Schmidtke-Schrezenmeier, Markus Urban, Anna Musyanovych, Volker Mailänder, Markus Rojewski, Natalie Fekete, Cedric Menard, Erika Deak, Karin Tarte, Volker Rasche, Katharina Landfester, Hubert Schrezenmeier

**Affiliations:** 1 DRK Blood Service Baden-Wurttemberg-Hessia, Institute for Clinical Transfusion Medicine and Immunogenetics Ulm and Institute of Transfusion Medicine, University of Ulm, Ulm, Germany; 2 Max-Planck-Institute for Polymer Research, Mainz, Germany; 3 INSERM U917, Universite Rennes 1, Faculte de Medecine, Rennes, France; 4 P6le Cellules et Tissus, CHU Pontchaillou, Rennes, France; 5 DRK Blood Service Baden-Wurttemberg-Hessia, Institute of Transfusion Medicine and Immuno haematology, Frankfurt, Germany; 6 University of Ulm, Department of Internal Medicine II, Ulm, Germany

## Abstract

*Background aims*. Mesenchymal stromal cells (MSC) are the focus of research in regenerative medicine aiming at the regulatory approval of these cells for specific indications. To cope with the regulatory requirements for somatic cell therapy, novel approaches that do not interfere with the natural behavior of the cells are necessary. In this context *in vivo* magnetic resonance imaging (MRI) of labeled MSC could be an appropriate tool. Cell labeling for MRI with a variety of different iron oxide preparations is frequently published. However, most publications lack a comprehensive assessment of the noninterference of the contrast agent with the functionality of the labeled MSC, which is a prerequisite for the validity of cell-tracking via MRI. *Methods.We* studied the effects of iron oxide-poly(L-lactide) nanoparticles in MSC with flow cytom-etry, transmission electron microscopy (TEM), confocal laser scanning microscopy (CLSM), Prussian blue staining, CyQuant® proliferation testing, colony-forming unit-fibroblast (CFU-F) assays, flow chamber adhesion testing, immuno-logic tests and differentiation tests. Furthermore iron-labeled MSC were studied by MRI in agarose phantoms and Wistar rats. *Results*. It could be demonstrated that MSC show rapid uptake of nanoparticles and long-lasting intracellular persistence in the endosomal compartment. Labeling of the MSC with these particles has no influence on viability, differentiation, clonogenicity, proliferation, adhesion, phenotype and immunosuppressive properties. They show excellent MRI properties in agarose phantoms and after subcutaneous implantation in rats over several weeks. *Conclusions*. These particles qualify for studying MSC homing and trafficking via MRI.

## Introduction

Mesenchymal stromal/stem cells (MSC) are the focus of interest in regenerative medicine, nurtured by their proven ability to differentiate into different cell types deriving from the mesoderm and their abundant availability because they are easy to culture and expand *in vitro* (1,2). Potential clinical applications are mainly in the field of bone, cartilage, skin, kidney and myocardial repair and immunomodulation (2-8). MSC can inhibit proliferation of T, B and natural killer (NK) cells and may interfere with function of dendritic cells (2). Because of their strong immunosuppressive potential, MSC also show promise for treatment of immunologic disorders (2). MSC are derived from different origins (bone marrow, adipose tissue, cord blood and others). They lack characterization by a unique, qualifying marker. The International Society of Cellular Therapy (ISCT) published a minimum set of criteria to define MSC (9); however, differences in MSC from different origins and in different culture conditions have been observed (10-13). Currently it is not known whether MSC act by differentiating into new tissue or by paracrine action, or a combination. Also, the optimal application mode and dose in different pathologies is under investigation (11-15). Studies on dose and biodistribution are important aspects of the assessment of the safety of MSC. They are considered to be an advanced therapy medicinal product (ATMP). The new European Union (EU) Directive 2009/120/EC amending Directive 2001/83/EC stipulates requirements for marketing and authorization of ATMP (16,17). This Directive requests data on ‘biodistribution, persistence and long-term engraftment of the somatic cell therapy medicinal product components’ (17). In this context *in vivo* tracking of MSC offers interesting opportunities, adding a new, non-invasive tool. Magnetic resonance imaging (MRI) is technically suitable to serve this need, but requires contrast labeling of the MSC administered. As the impact of changes in culture conditions and other manipulations on MSC is not fully elucidated yet, a contrast agent qualifying for MSC labeling *in vivo* must fulfill at least the following criteria *in vitro:* proven intracellular uptake and intracellular retention over time in quantities that change the MRI signal; no change of viability of the cells; no change in the set of MSC criteria as defined by ISCT; no alteration of MSC functionality; and a robust labeling procedure with only minimal interference with the *ex vivo* MSC expansion process.

The incorporation of different iron oxide-loaded particles in MSC and their MRI properties *in vitro* and *in vivo* has been shown by several groups (18-30). However, effects of iron-labeling on the biologic function, phenotype, differentiation potential and clonogenicity of MSC are controversially reported. Some groups report changes, whereas others do not see any differences underlining the importance of a comprehensive assessment for each labeling approach (31-34). Up to now, a systematic and comprehensive evaluation of the suitability of a specific MRI contrast agent/labeling technique for MSC labeling with regard to its lack of influence on MSC function is missing. Also, in most published studies data on kinetics, if provided at all, focus on uptake and do not provide detail on retention of the contrast agent. This information is essential for the interpretation of MRI data in long-term MRI observation studies, and important for defining the optimal labeling and labeled cell administration regimen. In the study presented here, we investigated MSC labeling with iron oxide-poly(l-lactide) (PLLA) nanoparticles synthesized via the mini-emulsion process (35,36). These nanoparticles have distinct advantages: PLLA is a polymer with a long history of safe use in medical applications and is fully biodegradable (37,38). The mini-emulsion process allows further introduction of different concentrations and types of iron oxide into the nanoparticles for optimization of the MRI properties, and also a fluorescent dye embedded in the polymer. The nanoparticles investigated have a negative zeta potential (between -29 and -44 mV) and a diameter of 110-135 nm.

Intracellular uptake and retention was evaluated quantitatively by flow cytometry and qualitatively by transmission electron microscopy, confocal laser scanning microscopy and Prussian blue staining. Expression of surface markers was determined by flow cytometry, and MSC differentiation into the osteo-blastic, adipogenic and chondrogenic lineages was induced and observed by standard methods. MSC functionality was tested by colony-forming unit-fibroblast assay (CFU-F), proliferation and MSC adhesion on human umbilical vein endothelial cells (HUVEC) cells under shear stress. MRI properties *in vitro* (agarose phantoms) and *in vivo* (white Wistar rats) were evaluated using a 3-Tesla clinical scanner.

## Methods

The nanoparticles were prepared with a mini-emulsion process (35). The nanoparticles tested were composed of poly(L-lactide) as a polymer and wuestite or magnetite as iron oxide (36). The particles were labeled with the fluorescent dye *N*-(2,6-diisopropylphenyl)-perylene-3,4-dicarbonacid-imide (PMI) (36).

### Materials

Ferric chloride hexahydrate (FeCl_3_-6 H_2_O; 99%; Merck, Darmstadt, Germany), oleic acid (58%; Riedel-de Haen, Seelze, Germany), methanol (98.5%; Merck), sodium hydroxide (99%; Merck), l-octadecene (92%; Merck), acetone (99%; Merck), *n*-octane (95%; Fluka, Buchs, Switzerland), Biomer®L9000 (number average molecular weight (*M*_n_)) *c.* 66 500 g/mol, weight average molecular weight (*M*_w_) *c.* 145 000 g/mol, determined by gel permeation chromatography (GPC) in chloroform; Biomer, Krailing, Germany), chloroform (99.99%; Fisher Scientific, Schwerte, Germany), sodium *n*-dodecyl sulfate (SDS; 99%; Alfa Aesar), PMI (BASF, Ludwigshafen, Germany) and hydrochloric acid (37%; AnalaR NORMAPUR; Prolabo, Leuven, Belgium). All chemicals were used as received. Demineralized (demin) water was used throughout the work.

### Synthesis of hydrophobized iron oxide nanoparticles

The synthesis was performed as described elsewhere (39). Briefly, NaOH (2.4 g) was dissolved in methanol (200 mL) and dropped into a solution consisting of FeCl_3_·6 H_2_O (5.4 g), oleic acid (17 mL) and methanol (100 mL). The brown precipitate obtained was washed five times with methanol and dried under reduced pressure. Afterwards, the brown solid was dissolved in l-octadecene (100 mL) at 70°C and 3 equivalent excess of oleic acid (for 25 nm wuestite particles) or 1 equivalent of oleic acid (for 10-25 nm magnetite particles) was added and the mixture heated to 300°C for 30 min under stirring in an argon atmosphere. By adding acetone:methanol at a 1:1 ratio, the iron oxide nanoparticles were precipitated and separated from the solution. The iron oxide nanoparticles were redispersed in *n*-octane and precipitated again by adding acetone:methanol and centrifuged again. The black residue obtained was dried at 40°C under reduced pressure.

### Preparation of poly(l-lactide) particles with encapsulated iron oxide

PLLA (300 mg), PMI (0.23 mg) and 150 mg of hydrophobized iron oxide nanoparticles were dispersed in chloroform (10 g) at 40°C and mixed afterwards with a solution consisting of water (24 g) and SDS (72 mg). After mechanical stirring for 1 h at 500 r.p.m., the mini-emulsion was prepared by ultra-sonication for 180 s (30-s pulse, 10-s pause) at 70% amplitude using a Branson sonifier W450 digital with a ½” tip under ice cooling, in order to prevent the evaporation of chloroform. The mini-emulsion was transferred into a round-bottomed flask with a wide neck and heated at 40°C under mechanical stirring (400 r.p.m.) overnight to evaporate the chloroform. The particles were purified to reduce the amount of surfactant and remove the non-encapsulated iron oxide and PMI. Therefore the sample was first centrifuged for 20 min at 420 *g* and then the upper phase was transferred into another tube. The sample was dialyzed (MWCO 100 000 membrane) by centrifugation for 30 min each at 690 *g* until the conductivity reached values below 9 μS/cm.

### Characterization of the particles

The particle size and zeta potential were measured using a Malvern Instruments Zeta Nanosizer with a detection angle at 173°, or a PSS NICOMP 380 Submicron Particle Sizer. The zeta potential was measured in a 10^−3^ m KC1 solution. For transmission electron microscopy, a Philips EM 400 or Zeiss EM 902 transmission electron microscope, both working at 80 kV, was used. Polymer particles were diluted with water, dropped on a carbon-coated 300-mesh copper grid, dried at ambient temperature and coated with carbon afterwards.

The amount of entrapped fluorescent marker was determined from the ultraviolet (UV)-visible absorption spectra of the particles. The measurements were carried out on an UV-visible spectrometer Lambda 16 from Perkin Elmer, Rodgau, Gemany; 5.6 mg of the freeze-dried sample was dissolved in 1 g chloroform. The iron oxide was decomposed using concentrated hydrochloric acid (37%) and the organic phase was washed afterwards three times with demin water. The chloroform phase was dried overnight under reduced pressure at 40°C and the solid was dissolved in the initial amount of chloroform afterwards. The absorbance of the solution was measured at 479 nm, which corresponded to a peak maximum for PMI. The amount of iron was determined by inductively coupled plasma-optical emission spectrometry (ICP-OES) with a Horiba Jobin Yvon Activa M. The sample was diluted (1:100) with a 0.75wt% SDS solution.

### Cultivation of human MSC

Cryopreserved *in vitro*-expanded human bone marrow-derived MSC of different passages were provided by the Institute of Transfusion Medicine of the University of Ulm (Ulm, Germany). Informed consent was obtained for collection of the original probe and covered by institutional review board (IRB) approval. Heparinized, unmanipulated bone marrow was seeded in cell culture flasks (Nunc, Roskilde, Denmark) in alpha-Minimum essential medium (MEM) (Lonza, Verviers, Belgium) and supplemented either by 20% fetal calf serum (FCS; Gibco fetal bovine serum; Invitrogen, Grand Island, NY, USA) or 10% human platelet lysate (PL; Institute for Clinical Transfusion Medicine and Immunogenetics, Ulm, Germany) (40). After 72-96 h, non-adherent cells were washed off and new medium was added. A medium exchange was performed weekly until cultures were almost confluent. Alpha-MEM with 10% PL (supernatant after centrifugation at 5000 r.p.m.) was supplemented with 12 μg/mL ciprofloxacin (Fresenius Kabi, Bad Homburg, Germany) and 2 IU/mL heparin (Ratiopharm, Ulm, Germany), in order to avoid clot formation and clumping of MSC. Alpha-MEM with 20% FCS was supplemented with 100 IU penicillin, 100 μg streptomycin (PenStrep Gibco; Invitrogen), 12 μg ciprofloxacin and 0.1 mg sodium pyruvate (Sigma, Munich, Germany) per mL medium. Cells were grown in a humidified incubator at 37°C and 5% CO_2_. MSC used in this study had shown osteogenic, chondrogenic and adipogenic differentiation.

For passaging/harvesting of the cells, medium was removed and cells were washed once with phosphate-buffered saline (PBS) and incubated with 0.5% trypsin (Invitrogen, Burlington, Canada) for 4-8 min at 37°C; detachment was checked visually before trypsin activity was neutralized with the addition of equal volumes of supplemented medium. For the experiments MSC were used between passages 1 and 10 (detailed information in the figure legends). Experiments that were repeated with MSC from different passages did not show significant differences of results. The number of population doublings of MSC that were used in the experiments ranged from 12.5 to 41.

### Prussian blue staining

Cytospins of MSC were prepared on glass slides. The slides were air dried for at least 30 min. Slides were fixed in methanol for 10 min and then incubated for 17 min in freshly prepared potassium hexacyanofer-rate (II) solution 2% w/w, with 0.1 N Hydrochloric acid (HCL), and washed in distilled water, counter-stained with hematoxylin-eosin for 5 min, washed three times in distilled water and air dried.

### Differentiation of human MSC

For the adiopogenic differentiation assay, adipogenic induction medium (PT-3102B; Lonza) was used. After 3-4 days, induction medium was removed and substituted by adipogenic maintenance medium for 3-4 days (PT3102A; Lonza). This cycle was repeated 3-4 times. Following the last cycle, cells were cultured for 3-4 days in maintenance medium. Then the cells were stained with Oil Red O.

For the chondrogenic differentiation assay, 1.5 mL NH ChondroDiff medium (130-091-679; Milentyi Biotec, Bergisch Gladbach, Germany) supplemented with 100 U penicillin and 100 μg streptomycin/mL medium was used. The differentiation medium was changed after 3 or 4 days. After 24 days, staining with methylene blue according to Loffler was done. MSC were washed with PBS, then methylene blue was added for 90 min. Staining solution was removed and acetous distilled water (six drops of acetatic acid in 100 mL distilled water) added for a few seconds. Then +4°C distilled water was added and slides were washed twice with distilled water.

For the osteogenic differentiation, 1.5 mL NH OsteoDiff medium (130-091-678; Miltenyi Biotec) supplemented with 100 U penicillin and 100 μg/mL medium streptomycin was used. The differentiation medium was changed every 3 or 4 days. At day 10, cells were Fast Blue® stained. For each differentiation assay, non-induced MSC were cultured as negative controls.

### Confocal laser scanning microscopy

Images were taken with Fluoview software on a Flu-oview 300 (Olympus, Hamburg, Germany) equipped with an 1X71 with two lasers, 488 and 543 nm, and a 60 × oil lens. PMI was excited by 488 nm laser light. For imaging of the cell membrane, 1 μL CellMask™Orange (Invitrogen, Grand Island, NY, USA) was added, which was excited by 543 nm laser light. Images were taken in the Kalman filter mode.

### Transmission electron microscopy

Cells were fixed with 2.5% glutaraldehyde containing 1.5% saccharose and 0.1 M phosphate buffer in PBS (pH = 7.^3^) and post-fixed in 2% aqueous osmium tetroxide. The samples were dehydrated in a 1-propanol series, block stained in 1% uranyl acetate and embedded in Epon. Ultra-thin sections were imaged in a Philips EM400 TEM, which was operated at a voltage of 80 kV.

### Flow cytometric analysis

For quantification of cellular particle uptake and determination of cell viability via 7-aminoactinomycin (7-AAD; Sigma Aldrich, St.Louis, MO, USA) staining, a FACSscan (Becton Dickinson) was used. Data were acquired and analyzed with Cell-quest 3.3 software (Becton Dickinson). Cells were washed, trypsinized and incubated for 15 min with 20 mg/mL 7-AAD and washed. FL1 was analyzed for nanoparticle uptake and FL3 for 7-AAD assessment. To allow comparison of uptake of different nanoparticles, FL1 values were normalized (nFL1). For this, the fluorescence signal was divided by the concentration of incorporated PMI in the particle. Surface antigens were analyzed with FACS-Aria® (Becton Dickinson). Commercial antibodies were used according to the recommendations of the manufacturers: CD3-Allophycocyanin (APC)-Cy7, CD9-APC, CD11b-phycoerythrin (PE), CD 14-PE, CD16-PE, CD19-PE-Cy7, CD29-PE, CD45-Peridinin Chlorophyll Protein Complex (PerCP), CD61-PerCP, CD71-APC, CD73-PE, CD90-PE, CD105-fluorescein isothiocyanate (FITC), CD166-PE, HLA-A, -B, -C-APC, anti-mouse IgG1-APC, anti-mouse IgG1-PE, anti-mouse IgG1-PE-Cy7, anti-mouse IgG1-PerCP, anti-mouse IgG1-APC-Cy7 (BD Biosciences, San Jose, CA, USA), CD 13-APC, CD105-APC (Caltag Labs, Burlingame, CA, USA), CD133-APC, CD271-APC (Miltenyi Biotec); HLA-DR-APC (R&D Systems, Minneapolis, MN, USA) and SSEA-4 -APC (eBioscience, San Diego, CA, USA).

### Adhesion under flow

The scope of this test was the analysis of MSC adhesion behavior under shear stress. HUVEC were seeded into μ slides (Ibidi Systems, Munich, Germany) in endothelial cell growth medium, as described previously (41). After reaching confluency, HUVEC were treated overnight with 100 ng/mL recombinant human tumor necrosis factor (TNF)-α (R&D Systems, Wiesbaden, Germany), which is known to induce expression of adhesion molecules on HUVEC and increase the number of adhered cells (41). MSC were trypsinized, dispersed in 37°C pre-warmed *N*_2_-hydroxyethylpiper-azine- *N*_2_-ethanesulfonic acid (HEPES)-buffered salt solution (HBSS) substituted with 1% human plasma, and kept at 37°C before use as a single-cell suspension. Within 60 min, 10^6^ MSC were flushed over the HUVEC at a calculated wall shear stress of 0.1 dynes/cm^2^, as described previously (41). After 5 min, when all MSC had passed the endothelial layer, numbers of adhered MSC were documented in representative fields using a charge-coupled device (CCD) camera; then the medium flow rate was increased to 2 dynes/cm^2^ for 5 min. After this, adherent cell numbers were determined again (41).

### CFU-F

To test clonogeneity, labeled and unlabeled MSC were seeded at different concentrations (3, 9, 18 or 54 cells/cm^2^) in standard alpha-MEM in six-well plates. After 14 days Giemsa staining was performed and the colonies were macroscopically and microscopically evaluated. A colony was defined as a concentric cell assembly > 50 cells.

### Proliferation test

A CyQuant®Cell proliferation assay (C7026; Invit-rogen, Paisley, UK) was performed. Cells were tryp-sinated and 200 cells/well were seeded per 24-well plate in 0.5 mL standard medium/well. In parallel, triple negative controls with 200 cells and 2000 cells were frozen at -80°C and stained and measured together with the cultivated cells. At day 7 optical microscopic judgments were made; samples were frozen at -80°C for at least 1 h. For staining and lysis, 19 mL distilled water were mixed with 1 mL of the kit buffer and 50 μL of the dye. Optical measurement was done via a multidetection microplate reader, Polarstar Omega (BMG Lab Tec, Offenburg, Germany).

### Assessment of immunosuppressive properties

Peripheral blood samples from healthy volunteers were provided by the French Blood Bank (EFS, Rennes, France). T and NK cells were purified using magnetic-negative cell selection kits (purity >97%; Miltenyi Biotech) before staining with 0.2 μM car-boxyfluorescein succinimidyl ester (CFSE; Interchim, Montlugon, France). MSC were labeled or not with nanoparticles at a final iron concentration of 100 μg/mL for 24 h and stimulated or not by 10 ng/mL interferon (IFN)-γ and 15 ng/mL TNF-α (R&D Systems) for 48 h. Supernatants were then harvested to assess indoleamine-2,3 dioxygenase (IDO) activity by measuring kynurenin concentration using high-performance liquid chromatography (HPLC) as described previously (42). CFSE-labeled T and NK cells were activated with 0.5 μg/mL anti-CD3 and anti-CD28 antibodies (Sanquin, Amsterdam, The Netherlands) or 100 IU/mL recombinant inter-leukin (IL)-2 (Novartis, East Hanover, NJ, USA), respectively. Labeled or unlabeled MSC were added at a 1:10 MSC:T and 1:1 MSC:NK ratios. Proliferation of CFSE^+^ Topro-3^−^ viable T and NK cells was assessed on day 5 of co-culture. The percentage of cells in each individual peak corresponding to cell generation was quantified using ModFit LT 3.0 software (Verity Software, Topsham, ME, USA).

### MRI

For phantom test samples, freshly prepared, cleared, liquid agarose 2% in PBS was placed in an agarose mold prepared 24 h before. Defined cell numbers diluted in 0.5 mL PBS were mixed thoroughly in liquid agarose specimens. For *in vivo* imaging, White Wistar rats with a weight of about 550-610 g were used, after obtaining regulatory approval according to the German animal protection act. Particle-labeled and unlabeled (negative control) MSC were surgically implanted subcutaneously, mixed in a collagen scaffold (Amedrix, Esslingen, Germany). A 3-Tesla clinical-grade MRI scanner with a sense flex coil (Achieva; Philips, Einhoven, The Netherlands) was used for imaging. *T*_1_ relaxation time (also called spinlattice or longitudinal relaxation time), *T*_2_ relaxation time (also called spin - spin relaxation time or transverse relaxation time) and the time constant for the observed decay of the free induction decay, i.e. the *T*_2*_ relaxation time was measured.

### Statistics

Each test was repeated at least twice and three independent samples were measured for each data point and evaluated by descriptive statistics, unless indicated otherwise. Data points represent the mean ± standard deviation unless indicated otherwise.

## Results

**Figure 1:**
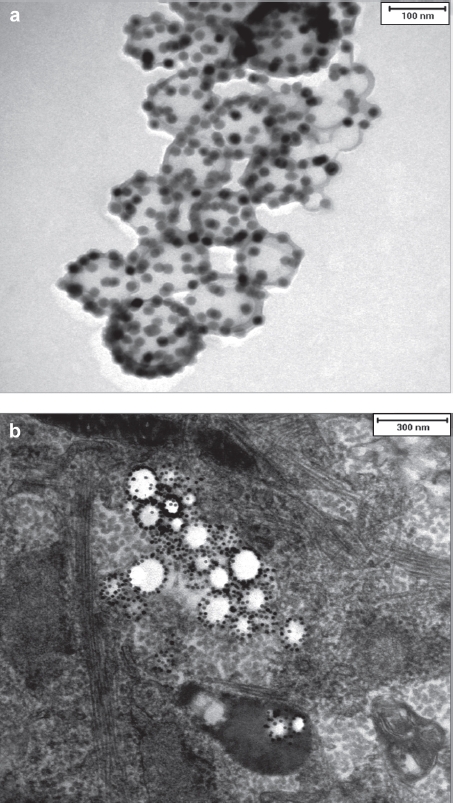
(a) TEM image of particle MU-Wuest 1 (the bar represents 100 nm); (b) representative example of a TEM image of MSC (passage 11) after 24 h incubation with MU-Wuest 1, showing endosomal particle agglomerates. The bar represents 300 nm.

For the experiments, different particle batches were used (MU-Wuest 1–4, with a particle diameter between 113 and 124 nm, a surface charge between -28 and -44 mV and an iron content ranging from 2.32 to 2.72 mg/mL). A transmission electron microscopy (TEM) image of particle MU-Wuest 1 is shown in Figure 1a.

For the intracellular uptake, persistence and tox-icity measurements, MSC were incubated for 24 h with nanoparticles and evaluated by TEM, confo-cal laser scanning microscopy (CLSM), fluorescent-activated cell sorting (FACS) and Prussian blue staining.

**Figure 2:**
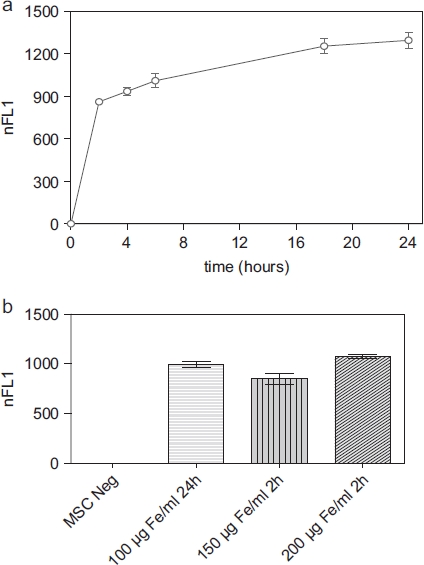
(a) FACS measurements showing the normalized relative fluorescence intensity (nFL1) of MSC (passage 5) incubated for 2, 4, 6, 18 and 24 h, respectively, with MU-Wuest 3 at a dose of 100 μg Fe/mL incubation medium. Results represent median ± standard deviation of triplicates, (b) FACS measurements showing the nFL1 intensity of MSC (passage 6) incubated for 24 h with MU-Wuest 3 100 μg Fe/mL or incubated for 2 h with 150 μg and 200 μg MU-Wuest 3, respectively. Results represent mean ± standard deviation of triplicates.

The nanoparticles showed excellent intracellular uptake, as demonstrated by TEM (Figure 1b), CLSM and FACS (Supplementary Figure 1a, b to be found online at: http://www.informahealthcare.com/cyt/10.3109/14653249.2011.571246). All MSC were labeled after incubation with the nanoparticles. Only minor nanoparticle attachments could be identified on the cell surface. Iron oxide-PLLA nanoparticles were taken up with a linear uptake increase with increasing doses, as shown by FACS measurements utilizing the fluorescent dye PMI included in the nanoparticles. Cellular uptake occurred rapidly within the first 2 h, reaching a plateau after 18 h (Figure 2a). By doubling the incubation concentrations, equal intracellular concentrations could be achieved after 2 h compared with half of the incubation concentration after 24 h incubation (Figure 2b).

**Figure 3:**
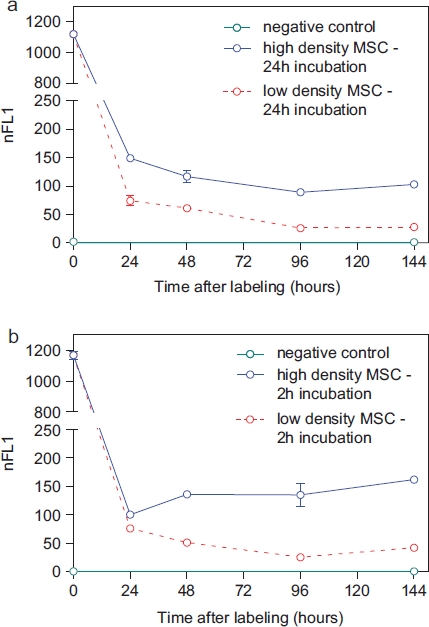
FACS measurements showing the nFL1 intensity of MSC (passage 5) incubated for 24 h with 100 μg Fe/mL (a) or incubated for 2 h with 200 μg Fe/mL MU-Wuest 3 (b) and then trypsinated and reseeded at high (20 000 cells/cm^2^) and low (5000 cells/cm^2^) densities. FACS measurements were done directly after incubation (0 h) and 24, 48, 96 and 144 h after particle removal and reseeding. Results represent mean ± standard deviation of triplicates. Most of the standard deviations are too small to be seen in this graph.

**Figure 4:**
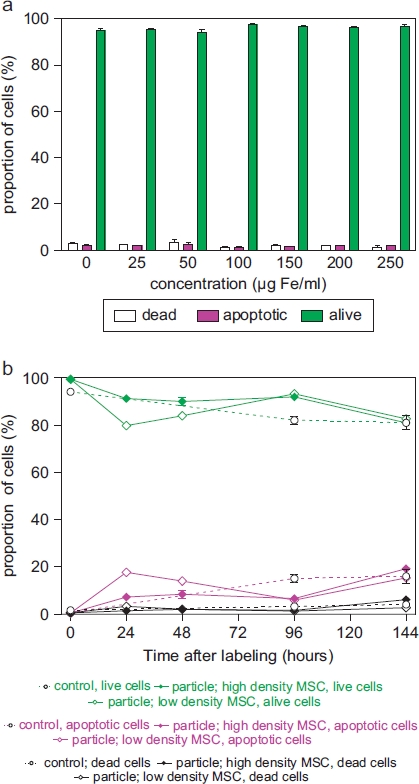
Relative proportion of living, apoptotic and dead cells as assessed by FACS measurements of 7-AAD-stained (a) MSC (passage 8), which were incubated for 24 h with MU-Wuest 3 in doses from 25 μg Fe/mL up to 250 μg Fe/mL, and (b) MSC incubated for 24 h with MU-Wuest 3 (100 μg Fe/mL) and then reseeded (5 × 10^3^ and 2 × 10^4^ cells/cm^2^) and followed-up 144 h after reseeding. Results represent mean ± standard deviation of triplicates. Dotted lines represent unlabeled control cells.

**Figure 5:**
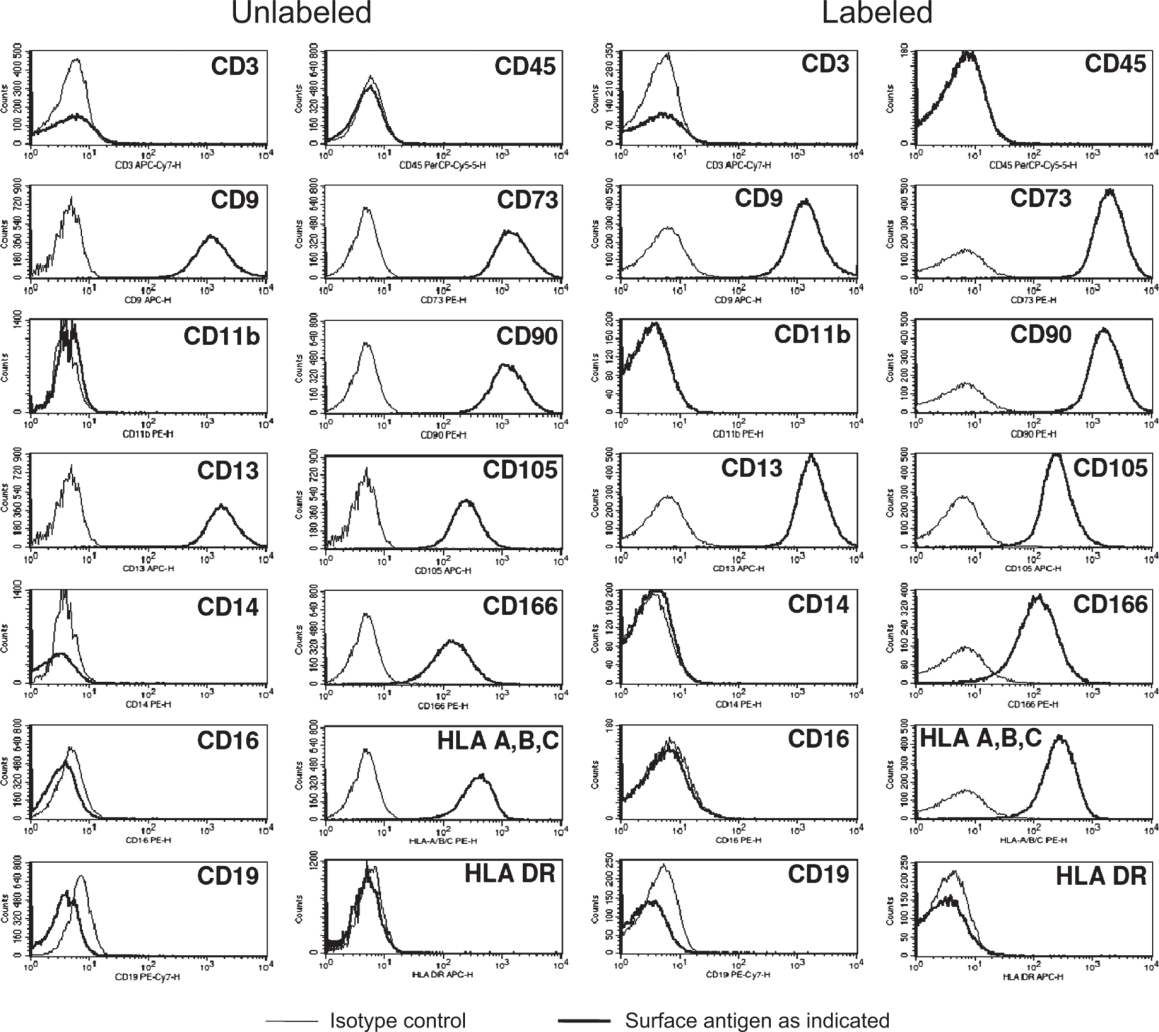
Expression of CD45, CD3, CD19, CD14, CD16, CD19, CD11b, CD9, CD13, CD73, CD90, CD105, CD166, HLA-A, -B, -C and HLA-DR on unlabeled control MSC and MSC 48 h after labeling with MU-Wuest 3 (passage 4 MSC). Thin lines show isotype controls and bold lines the expression of the respective surface antigen.

Intracellular nanoparticle persistence was demonstrated for up to at least 14 days after iron oxide-PLLA particle removal with Prussian blue staining (Supplementary Figure 2 to be found online at: http://www.informahealthcare.com/cyt/10.3109/14653249.2011.571246), up to at least 8 days with TEM imaging and up to at least 6 days with FACS measurements. This did not preclude the particles persisting in the cells even longer. Directly after incubation, high intracellular nanoparticles levels were reached. Within 24 h of particle removal, intracellular nanoparticle levels dropped sharply and thereafter showed slowly descending intracellular levels (Figure 3a, b). We wondered whether intracellular persistence might differ between labeling protocols with a short incubation of high concentrations and vice versa. We found that there was no difference in intracellular persistence between the 2 h/200 μg Fe and 24 h/100 μg Fe labeling protocols (Figure 3a,b). Reseeding of the labeled cells at a low density (5 × 10^3^ cells/cm^2^) resulted in lower intracellular particle persistence in comparison with high-density seeded (2 × 10^4^ cells/cm^2^) labeled MSC. As there was less contact inhibition and more proliferation under the conditions of 5 × 10^3^ cells/cm^2^, this could indicate a distribution of the particles to daughter cells during cell division (Figure 3). No short- or long-term impact of iron oxide-PLLA nanoparticle labeling on MSC viability, as determined by FACS measurements and 7-AAD staining, could be identified (Figure 4). No influence of the iron oxide-PLLA nanoparticle labeling on the MSC adi-pogenic, chondrogenic and osteogenic MSC differentiation potential could be detected (Supplementary Figure 3 to be found online at: http://www.infor-mahealthcare.com/cyt/10.3109/14653249.2011.571246). No influence of iron oxide-PLLA particle labeling on the typical surface antigen pattern, as defined by ISCT, could be found (Figure 5). Differences between labeled and unlabeled cells could be identified with regard to CD71 (transferrin receptor). Whereas with the unlabeled MSC, CD71 expression increased after reseeding, in the iron oxide-PLLA particle-labeled MSC, CD71 expression decreased following reseeding after labeling and then returned to the basic value at day 14 (Supplementary Figure 4 to be found online at: http://www.informahealthcare.com/cyt/10.3109/14653249.2011.571246).

No influence of labeling on the clonogenicity as evaluated by CFU-F assay could be observed (Supplementary Figure 5 to be found online at: http://www.informahealthcare.com/cyt/10.3109/14653249.2011.571246). The influence of iron oxide-PLLA nanoparticle labeling on MSC proliferation was tested using a CyQuant® assay. No influence of particle labeling on the proliferation behavior of the MSC could be discovered (Supplementary Figure 6 to be found online at: http://www.informahealthcare.com/cyt/10.3109/14653249.2011.571246).

**Figure 6:**
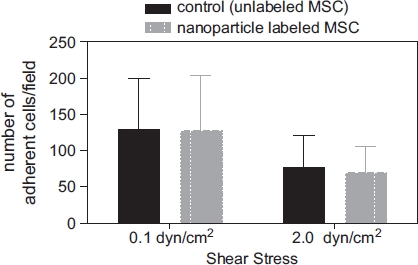
MSC adhesion under shear stress showing the number of adherent cells on a HUVEC cell layer pre-stimulated with TNF-α. MSC were labeled with MU-Wuest 3 at 100 μg Fe/mL for 24 h and thereafter tested. MSC were adhered to HUVEC under flow at 0.1 dynes/cm^2^. The resistance of the adhered cells to increased shear stress was determined after raising the shear stress to 2.0 dynes/cm^2^. Results indicate mean ± standard deviation of a total of six experiments using three different MSC lines (passages 4, 6 and 10).

The influence of iron oxide-PLLA nanoparticle labeling on adhesion of MSC was studied in a test model where the adhesion capability of MSC to a HUVEC cell layer, which had been pre-stimulated by TNF-α, was determined under shear stress. Once again, no difference between labeled and unlabeled cells could be found, both in the rate of initial accumulation and the resistance to increased shear stress (Figure 6).

**Figure 7:**
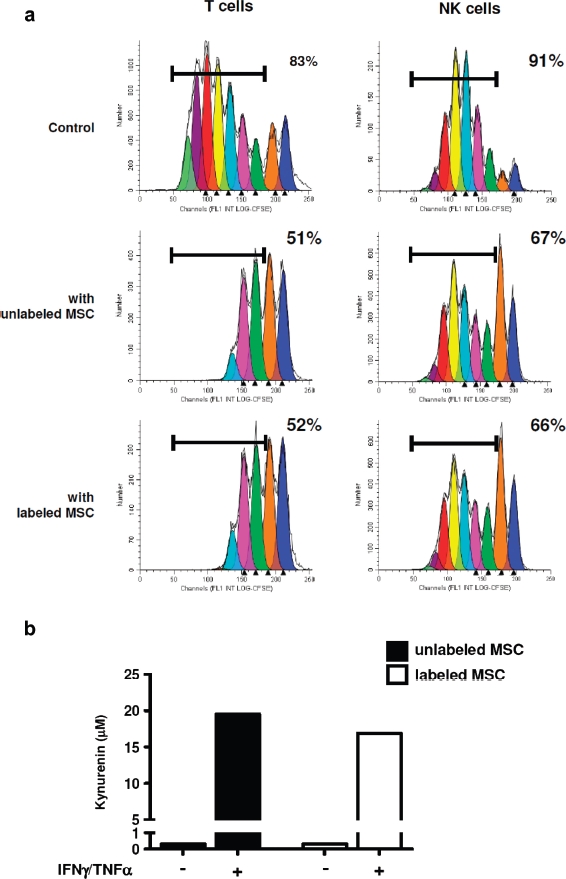
(a) Proliferation of purified T and NK cells in response to CD3/CD28 cross-linking or IL-2, respectively. CFSE dilution was evaluated on day 5 of culture without MSC (upper panels) or in the presence of unlabeled (middle panels) or labeled (lower panels) MSC. MSC (passage 1) were labeled with MU-Wuest 4 100 μg Fe/mL for 24 h. Results are expressed as the percentage of T or NK cells that had undergone more than one cell division; one representative experiment of two. (b) MSC previously labeled or not with nanoparticles were treated with IFN-γ/TNF-α for 2 days and IDO activity was assessed by quantification of kynurenin in cell supernatants; one representative experiment of two.

The role of MSC as modulators of immune responses is crucial for their clinical potential, making it mandatory to check that this immunosuppressive function is preserved after iron-labeling. Importantly, the capacity of MSC to inhibit both T and NK cell proliferation was not altered by nanoparticle labeling (Figure 7a). In addition, we confirmed that conditioning of MSC by a combination of IFN-γ/TNF-α reinforced their immunosuppressive properties and demonstrated that the capacity of MSC to respond to these inflammatory stimuli was not modified after nanoparticle labeling, as revealed by their increased capacity to inhibit both T and NK cell proliferation (Supplementary Figure 7 to be found online at: http://www.informahealthcare.com/cyt/10.3109/14653249.2011.571246). In agreement, whereas iron-labeling of MSC did not promote IDO activity by itself, labeled and unlabeled MSC displayed a similarly high IDO activity in the presence of IFN-γ and TNF-α (Figure 7b).

It has been reported that intracellular iron oxide shows a pronounced *T*_2*_ effect (22) and that the difference of *T*_2_/*T*_2*_ may even help to discriminate extracellular from intracellular iron oxide (43). Agarose phantoms prepared with different particle concentrations showed that the native iron oxide-PLLA particles already exerted a pronounced *T*_2*_ effect and only a weak *T*_2_ effect in contrast to an equal *T*_2_/*T*_2*_ effect of a carboxydextran-coated, formerly commercially available, iron oxide contrast agent (Resovist®; Bayer-Schering-Pharma), indicating that the steric arrangement of the iron oxide maybe the main contributor to this effect. This was also reflected in the relaxivity values *r*_2_ and *r*_2*_ (1/*T*_2_, 1/*T*_2*_, respectively; Supplementary Table I to be found online at: http://www.informahealthcare.com/cyt/10.3109/14653249.2011.571246). Also in the agarose phantoms with iron oxide-PLLA nanoparticle-labeled MSC, *T*_2*_ showed the most pronounced effect.

**Figure 8:**
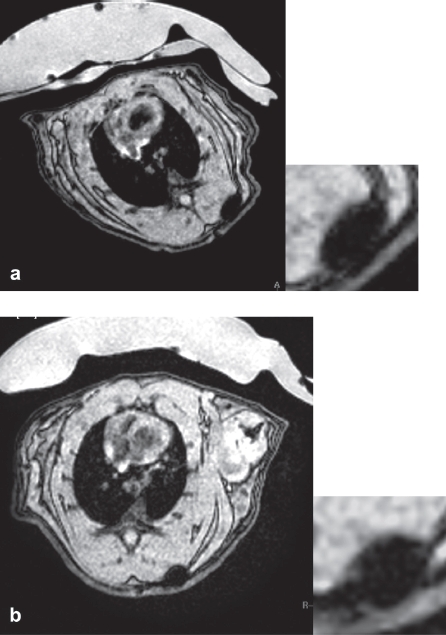
1 × 10^6^ MSC (passage 4) labeled with MU-Wuest 4 100 μg Fe/mL in 1 mL of a collagen scaffold 2 × 3 × 2-mm subcutaneous implant, (a) MRI taken 2 days after implantation; (b) MRI 25 days after implantation. As a control, the same number of unlabeled MSC in a scaffold was implanted on the contralateral side. No *T*_2_/*T*_2*_ signal could be detected at the implantation site of the control.

To investigate whether MRI data matched the FACS data with regard to cellular particle retention, an agarose phantom was prepared loaded with samples of 2 × 10^5^ or 5 × 10^4^ MU-Wuest 3-labeled cells/mL agarose, directly after labeling and 24 and 96 h after reseeding with labeling at high (2 × 10^4^ cells/cm^2^) or low (5 × 10^3^ cells/cm^2^) densities, respectively. Images matched the FACS data, showing the strongest T_2_* signal directly after labeling in the 2 × 10^5^ cell/mL sample, with decreasing signal strength at 24 and 96 h. An identical finding applied for the 5 × 10^4^ cells/mL sample. A signal difference could also be seen between the high and low density-seeded cell samples, with the low density showing a weaker signal than the high density-seeded cells (Supplementary Figure 8 to be found online at: http://www.informahealthcare.com/cyt/10.3109/14653249.2011.571246). Signal quantification matched the optical findings and thus again showed consistency with the FACS data. *In vivo* imaging properties were tested by implanting 1 × 10^6^ PLLA particle-labeled human MSC, mixed in a 1-mL collagen scaffold in 3 × 2 × 3-mm pieces, subcutaneously in a rat. The implant was followed-up with multiple MRI sessions up to 25 days after implantation. The implant showed an excellent signal with no deterioration over time, indicating that the cells did not migrate out of the scaffold (Figure 8). Histologic evaluation of the implanted scaffolds stained with HE and Prussian blue showed spindle-shaped cells with cytoplasmatic iron, located exclusively in the collagen scaffold. This finding was consistent with the MRI observation. In another rat it could be shown that 0.8 × 10^6^ MSC/mL collagen gel implanted 24 h after particle removal (i.e. having a much lower particle load according to the kinetic experiments) still showed good visibility after implantation, with a decreasing and finally vanishing signal at 35 days after implantation.

## Discussion

Iron labeling of MSC for MRI is a frequently published procedure, however most publications focus on short-term uptake and follow-up *in vivo* and on the basic assessment of differentiation capability. To exploit the promise and potential of this method fully, for regulatory purposes on somatic cell therapeutics and for correct interpretation of *in vivo* imaging data, more knowledge on the subcellular distribution, retention of particles in cycling and quiescent cells and particle influence on the MSC phenotype and functionality, is necessary. In the past the focus has been on super-paramagnetic iron oxides (SPIO) already approved for use in humans. However, in most MSC-labeling regimens SPIO are to be used together with transfection agents for efficient intracellular labeling and, if used without, relevant amounts of the SPIO stick on the surface of the cells (19-21,26,44). One of the most suitable ones, Resovist®, was recently removed from the market, thus stressing a demand for an innovative, non-interfering MRI contrast agent for cell labeling. Uptake kinetics of nanoparticles depends on the polymer, surface charge, surface functionalization and size of the particle. Whereas some particles are taken up slowly and reach their maximum uptake only after 24 h or even later, for example polystyrene-based particles (24), others are taken up very rapidly, for example polyisoprene particles, which reach a plateau after approximately 4 h of incubation (25). Compared with this, the PLLA particles studied here show a rather fast uptake, with about a 70% uptake reached after 2 h and a slow increase of label intensity up to 24 h (the last time-point measured).

The uptake kinetics of the PLLA particles studied in our studies is very similar to the uptake pattern of PLLA or poly(ε-caprolactone) particles into HeLa cells (45). It is notable that the dose-uptake correlation did not show saturation up to the highest concentration tested. This is in agreement with saturation kinetics of other nanoparticles synthesized by the mini-emulsion process, such as PBCA particles (46) and polyisoprene particles (25).

The uptake of nanoparticles can happen via various endocytotic mechanisms (47). Like many other particles, the iron oxide-PLLA particles end up in endosomes (21,47,48). The results on intracellular particle persistence are consistent with published literature, where different iron-labeling regimens have been used (22,23,49-52).

There are publications reporting an influence of iron labeling on MSC biology. For iron labelling additional transfection agents are frequently necessary. These transfection agents might influence MSC biology (31,33). The goal of iron labeling MSC is to gain more insight into the *in vivo* behavior of MSC. Iron oxide-PLLA particles have been developed recently (36). They have the advantage of full biodegradability of the polymer (38). Our investigation demonstrated undisturbed cell viability, even with high particle concentrations, and a long-lasting intracellular retention that is sufficient for MRI detection over a prolonged period. We could demonstrate that trifunctional differentiation is not influenced by labeling the MSC with iron oxide-PLLA particles. As MSC adhesion is very relevant for MSC homing (41), we compared the adhesion capability between labeled and unlabeled cells under shear stress and could not detect any differences. The lack of influence on the clonogenicity of the MSC was verified via CFU-F assays, and an influence on the proliferative capabilities of MSC could not be demonstrated by proliferation assays. The MSC phenotype remained unchanged with regard to the basic criteria as defined by the ISCT (9). A difference between labeled and unlabeled cells was only observed for the expression of CD71, the transferrin receptor. CD71 showed a transient difference between labeled and unlabeled cells. In accordance with our results, others have shown that ferumoxides protamine sulfate complexes also result in a transient decrease of transferrin receptor mRNA and protein (34). In contrast, others have reported that Resovist® without a transfection reagent causes an enhanced expression of CD71 (53). Whether our finding is of any relevance remains to be elucidated. CD71 plays a role in transferrin-bound iron uptake (54). It is over-expressed in highly proliferating tissues (55,56) and is not expressed in immature progenitor cells (56-58). CD71 is an endosomal-associated protein that is recycled to the plasma membrane after release of iron (59,60). The reason for the lack of up-regulation could be assumed to be the iron loading of the cell or the interference of the particle with the endosomal receptor recycling pathway. As different iron oxide-containing preparations (e.g. Resovist®, PLLA nanoparticles and ferumoxides protamine sulfate) might differ in intracellular trafficking and metabolism, the release of iron and the influence on iron metabolism might also differ.

The immunomodulatory potential of MSC was assessed by inhibition of T and NK cell proliferation and by production of functional IDO, an immunosup-pressive mechanism consistently reported for human MSC. No difference could be observed between labeled and unlabeled MSC for these parameters, indicating that the MSC behavior towards immune cells and response to inflammatory cytokines was unaffected by nanoparticle labeling. This is of particular interest as the immunosuppressive properties of MSC are usually triggered by inflammatory signals and are crucial for their *in vivo* efficacy.

The suitability of the particles for *in vivo* imaging has also been demonstrated by the first *in vivo* studies in rats. T_2_* qualified as lead parameter in the MRI quantification, with clear dose- and time-dependency and clear differences between labeled and unlabeled cells in the agarose phantom. This is in line with the literature, where *T*_2_* is described as the most sensitive parameter for intracellular iron (43,61,62). We showed that the iron oxide-PLLA-labeled MSC are easily detectable in MRI *in vitro* and *in vivo* and thus present a promising tool for elucidating the function of MSC and optimization of an application mode in addition to homing and trafficking *in vivo* by non-invasive MRI, thus also fulfilling the regulatory requirements for data on biodistribution of MSC.

In conclusion, it was demonstrated that iron oxide-PLLA particles are promising candidates for MSC labeling for MRI because they did not change the MSC biology in the comprehensive *in vitro* test settings applied, and showed excellent MRI properties over several weeks, as demonstrated in rats. This method could also help us gain more insight not only regarding the homing and trafficking of MSC but also in their mode of action in different indications, and provide relevant guidance for the optimal application mode of MSC in different indications.
