# Supplementary material for: Labeling of mesenchymal stromal cells with iron oxide-poly(l-lactide) nanoparticles for magnetic resonance imaging: uptake, persistence, effects on cellular function and magnetic resonance imaging properties
Source: Cytotherapy. 2011 Apr 15;13(8):962–75. doi: 10.3109/14653249.2011.571246 (PMC3172145; doi:10.3109/14653249.2011.571246)
Supplement: Supplementary Material [file 571246_suppl.pdf]

Supplementary material for Schmidtke-Schrezenmeier G, Urban M, Musyanovych A, Mailänder V, Rojewski M, Fekete N, Menard C, Deak E, Tarte K, Rasche V, Landfester K & Schrezenmeier H. Labeling of mesenchymal stromal cells with iron oxide–poly(L-lactide) nanoparticles for magnetic resonance imaging: uptake, persistence, effects on cellular function and magnetic resonance imaging properties. Cytotherapy. 2011; 13: 962–975.

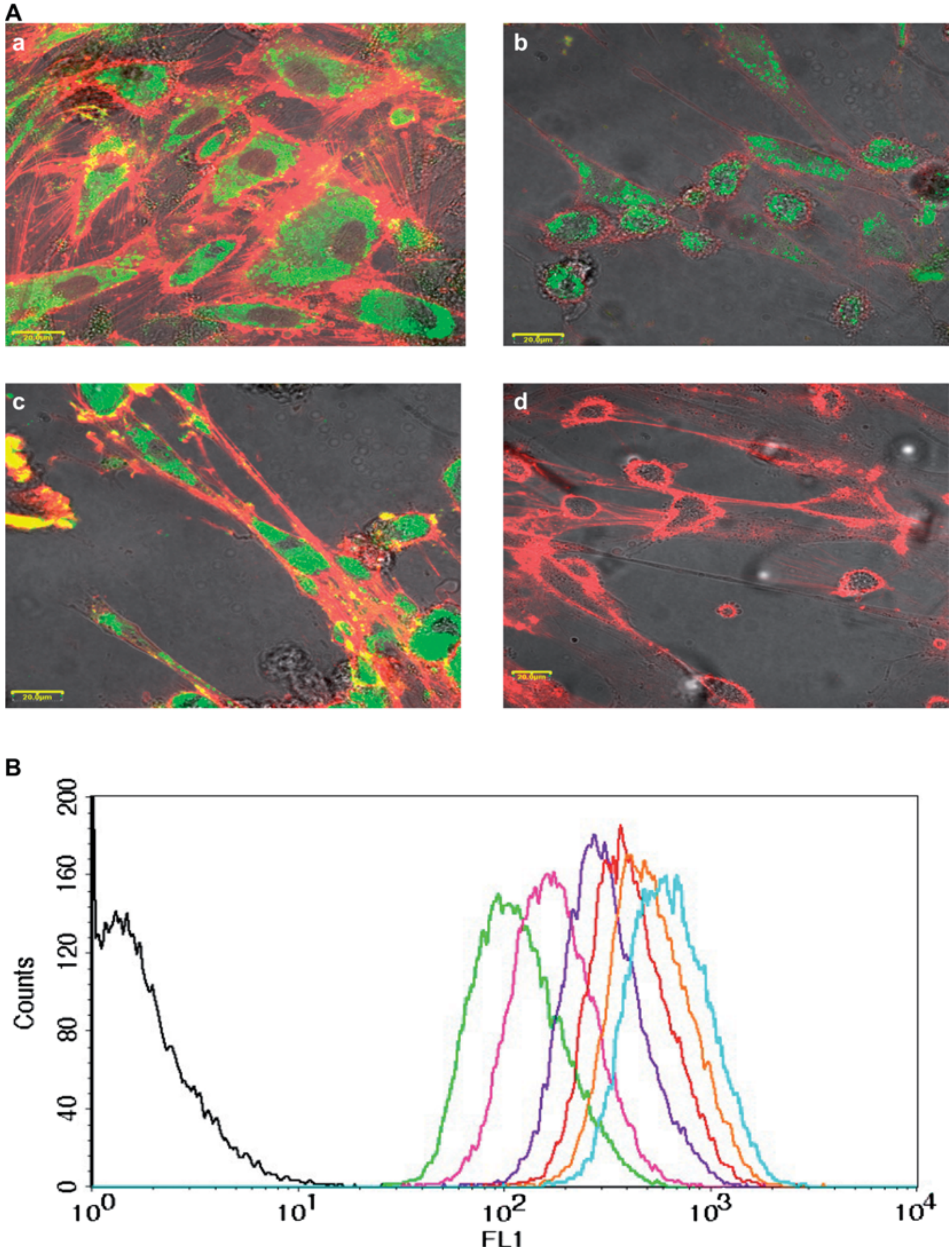

Supplementary Figure 1. A: Representative CLSM images of MSCs (passage 5) incubated 24 h with MU-Wuest2, MU-Mag1 and MU-Wuest1 in a concentration of 100 µg Fe/ml. Images show intracellular location of iron-PLLA particles with only minor cell surface particle aggregates. Red: cell membrane stained with CellMask; green: PMI included in the PLLA of the particles; yellow: overlay of green and red indicating particle located at extracellular side of cell membrane; a) MSCs labeled with MU-Wuest2; b) MSCs labeled with MU-Mag1; c) MSCs labeled with MU-Wuest1, and d) MSC negative control. B: FACS fluorescence intensities of MSC (passage 8) after labeling with MU-Wuest2 in doses of 25 µg Fe/ml to 250 µg/ml. From left to right: black: negative control; green: 25 µg Fe/ml; purple: 50 µg Fe/ml; blue: 100 µg Fe/ml; brown: 150 µg Fe/ml; orange: 200 µg Fe/ml; turquoise: 250 µg Fe/ml.

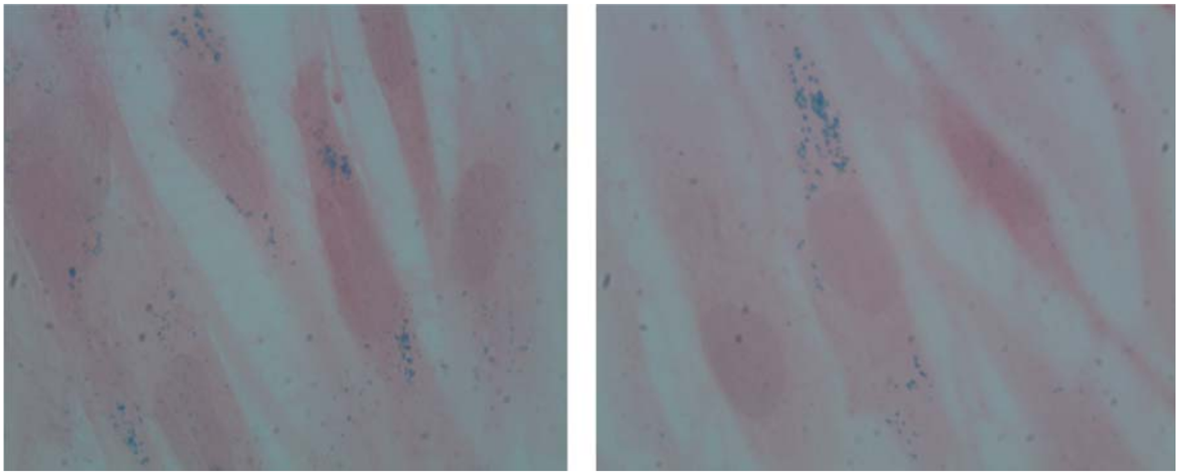

Supplementary Figure 2. Representative example of prussian blue staining of MSC 14 days after particle removal. MSCs were incubated 24 h with MU-Wuest3, washed and trypsinated and re-seeded in a density of 54 cells/cm^2.

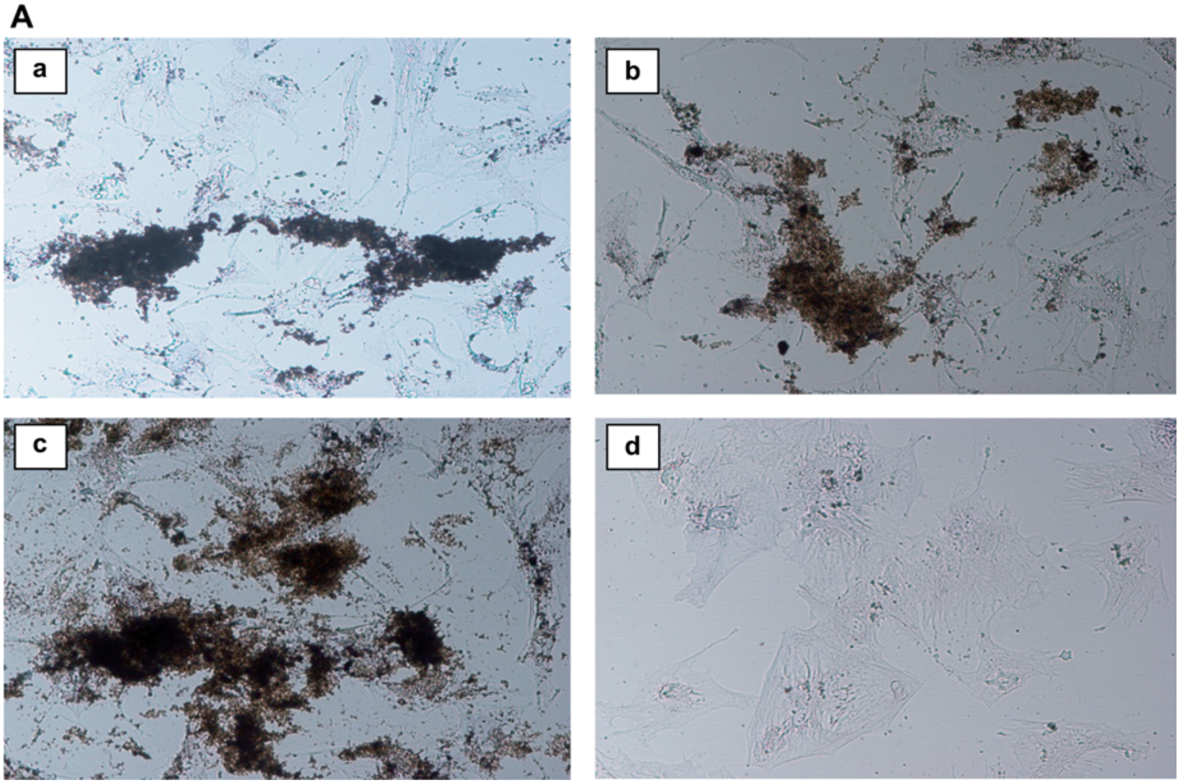

Supplementary Figure 3. A: Osteocyte differentiation test with MSCs (passage 10): Images show the differentiation – black (Sigma Fast®B5655 stained) is the alkaline phosphatase produced by the osteocytes; magnification 10x. a) negative control – unlabeled MSCs but otherwise treated identically with differentiation medium; b) MSCs incubated 24 h with MU-Wuest3 (50 µg Fe/ml); c) MSCs incubated 24 h with MU-Wuest3 (250 µg Fe/ml); d) negative control – unlabeled MSCs not treated with differentiation medium; B: Adipocyte differentiation test with MSCs: Images show the differentiation. The red droplets (Oil Red O Stain) indicate the intracellular lipid vacuoles; magnification 10x; a) negative control – unlabeled MSCs but otherwise treated identically with differentiation medium; b) MSCs incubated 24 h with MU-Wuest3 (50 µg Fe/ml); c) MSCs incubated 24 h with MU-Wuest3 (250 µg Fe/ml); d) negative control – unlabeled MSCs not treated with differentiation medium; C: Chondroblast differentiation test with MSC: Images show the differentiation. The blue fibrillary structures (Methylenblau according to Löffler stained) indicate the collagen fibrilles of the chondroblasts; magnification 10x; a) negative control – unlabeled MSCs but otherwise treated identically with differentiation medium; b) MSCs incubated 24 h with MU-Wuest3 (50 µg Fe/ml); c) MSCs incubated 24 h with MU-Wuest3 (250 µg Fe/ml); d) negative control – unlabeled MSCs not treated with differentiation medium

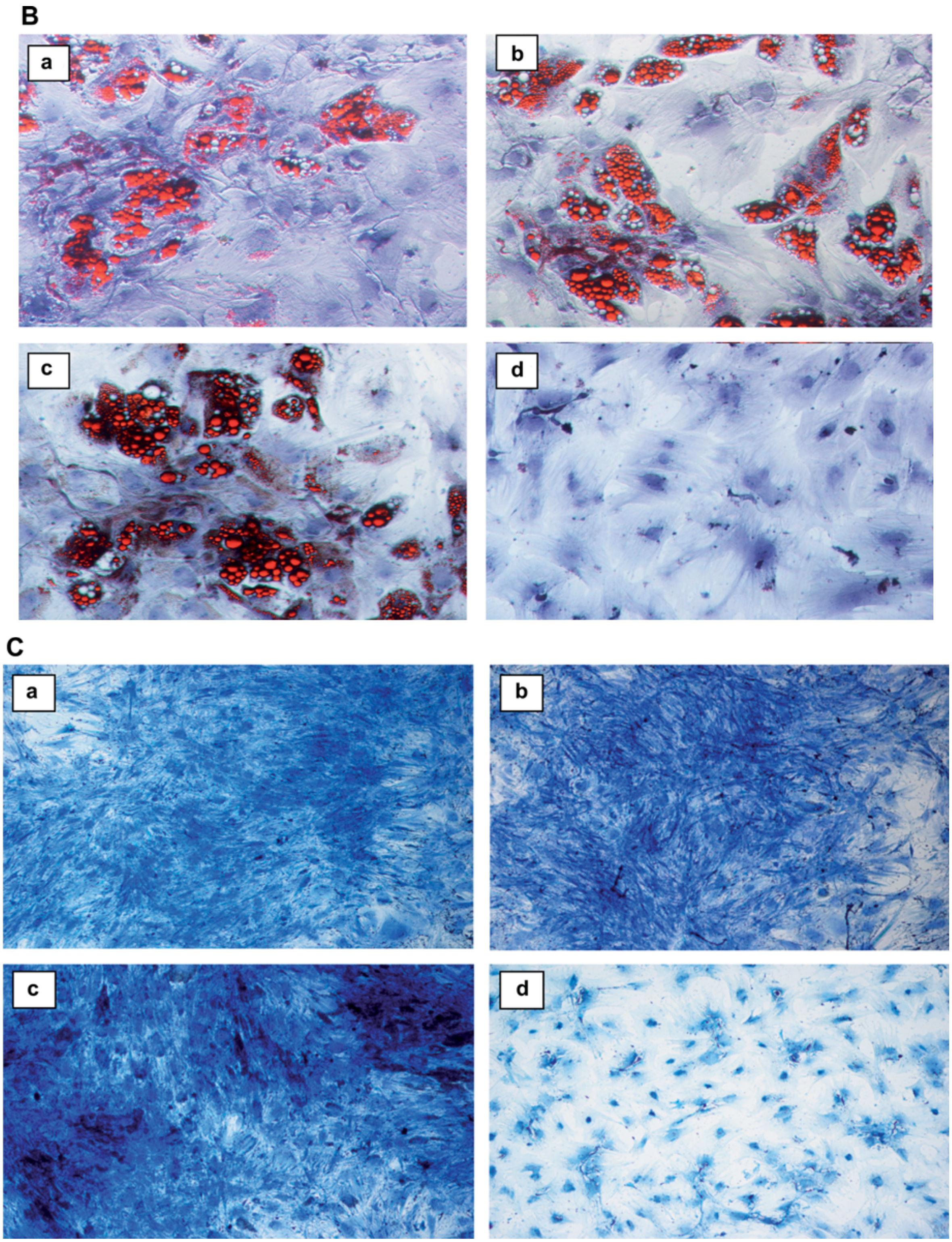

Supplementary Figure 3. (*Continued*).

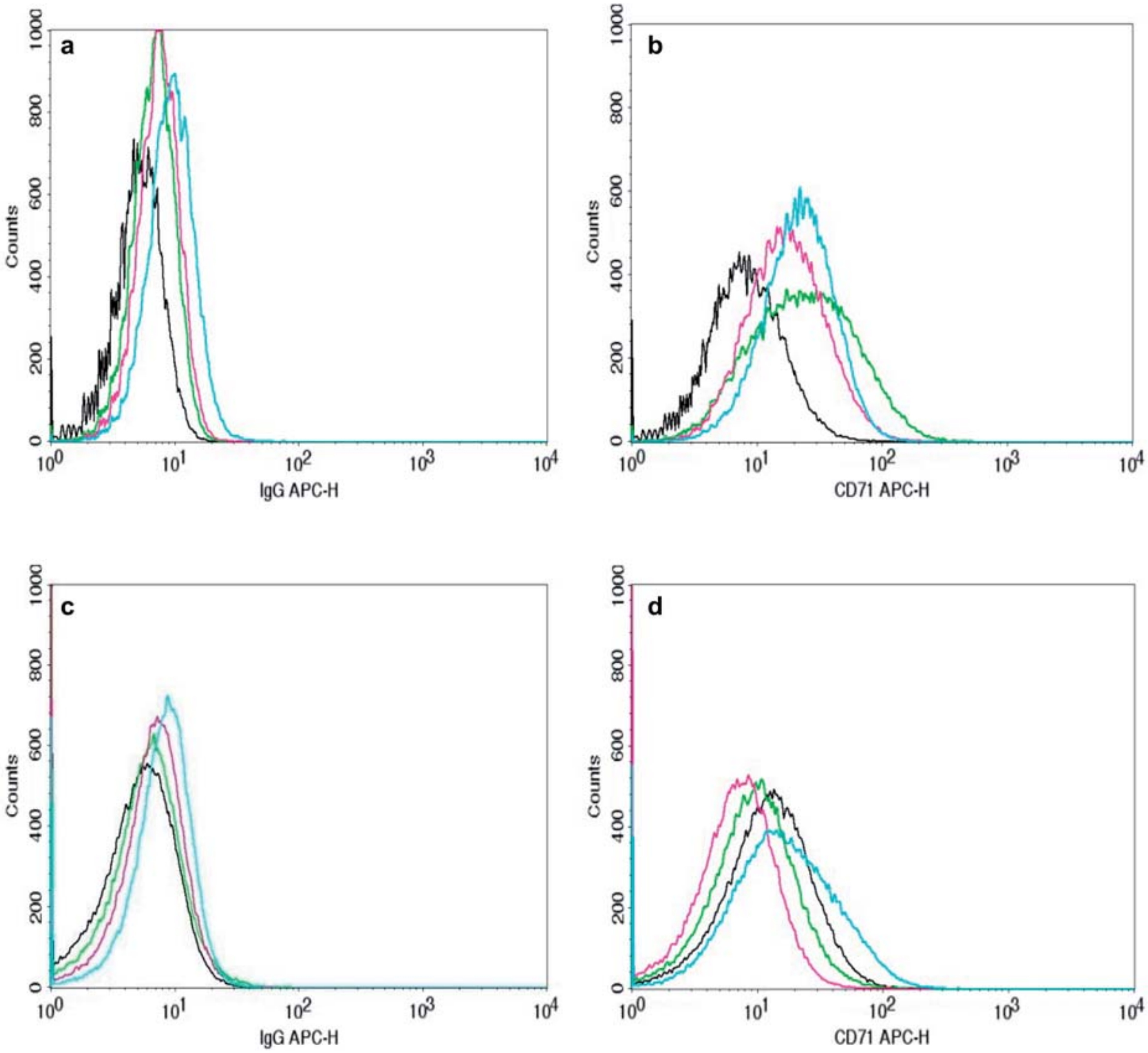

Supplementary Figure 4. Representative example of FACS curves showing CD71 expression of MSC (passage 4) on day 0, 2, 4 and 14 after MU-Wuest3 removal and re-seeding;black: d0; green:d2; red: d6; blue: d14 a) isotype control negative control, i.e. unlabeled MSC; b) CD71 expression negative control; c) isotype control MU-Wuest3 labeled cells; d) CD71 expression MU-Wuest3 labeled cells;

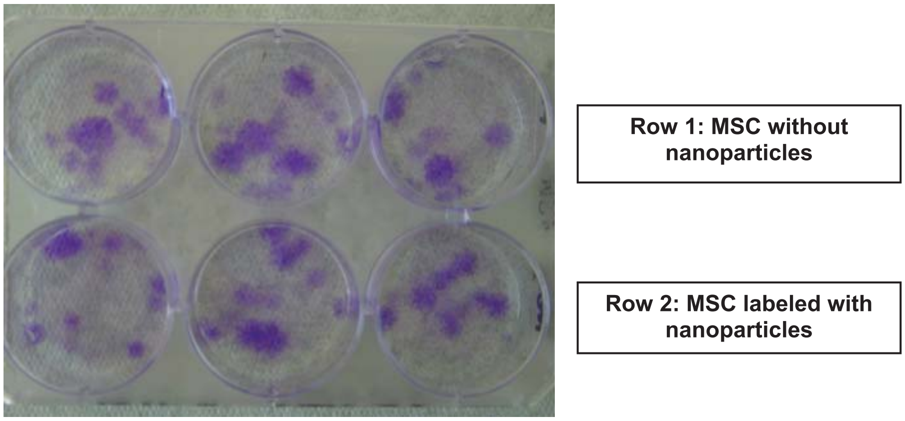

Supplementary Figure 5. CFU-F assay with MSCs incubated with 100 µg Fe/ml MU-Wuest3 and unlabeled MSCs (passage 4), respectively. Seeding concentration: 3 cells/cm^2.

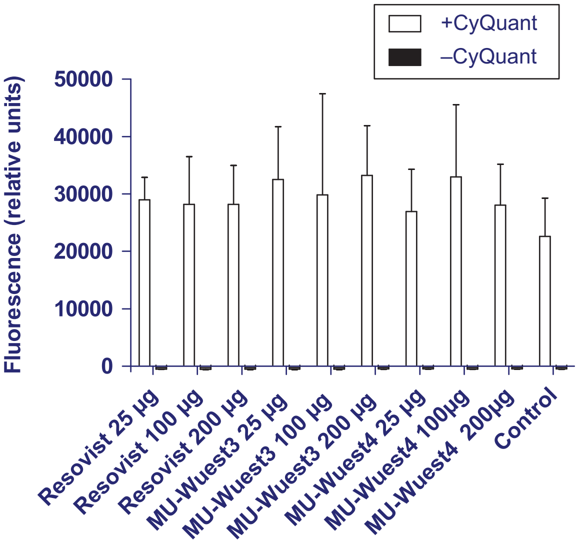

Supplementary Figure 6. Proliferation test showing the proliferative activity of 3 different MSC cell lines (passages 3-5) after incubation with 25, 100 and 200 µg Fe/ml of MU-Wuest3, MU-Wuest4 particles or Resovist®/Poly-L-Lysin. The incubation concentration indicates the iron oxide concentration. Values represent mean ± standard deviation of 3 experiments (performed in triplicate each).

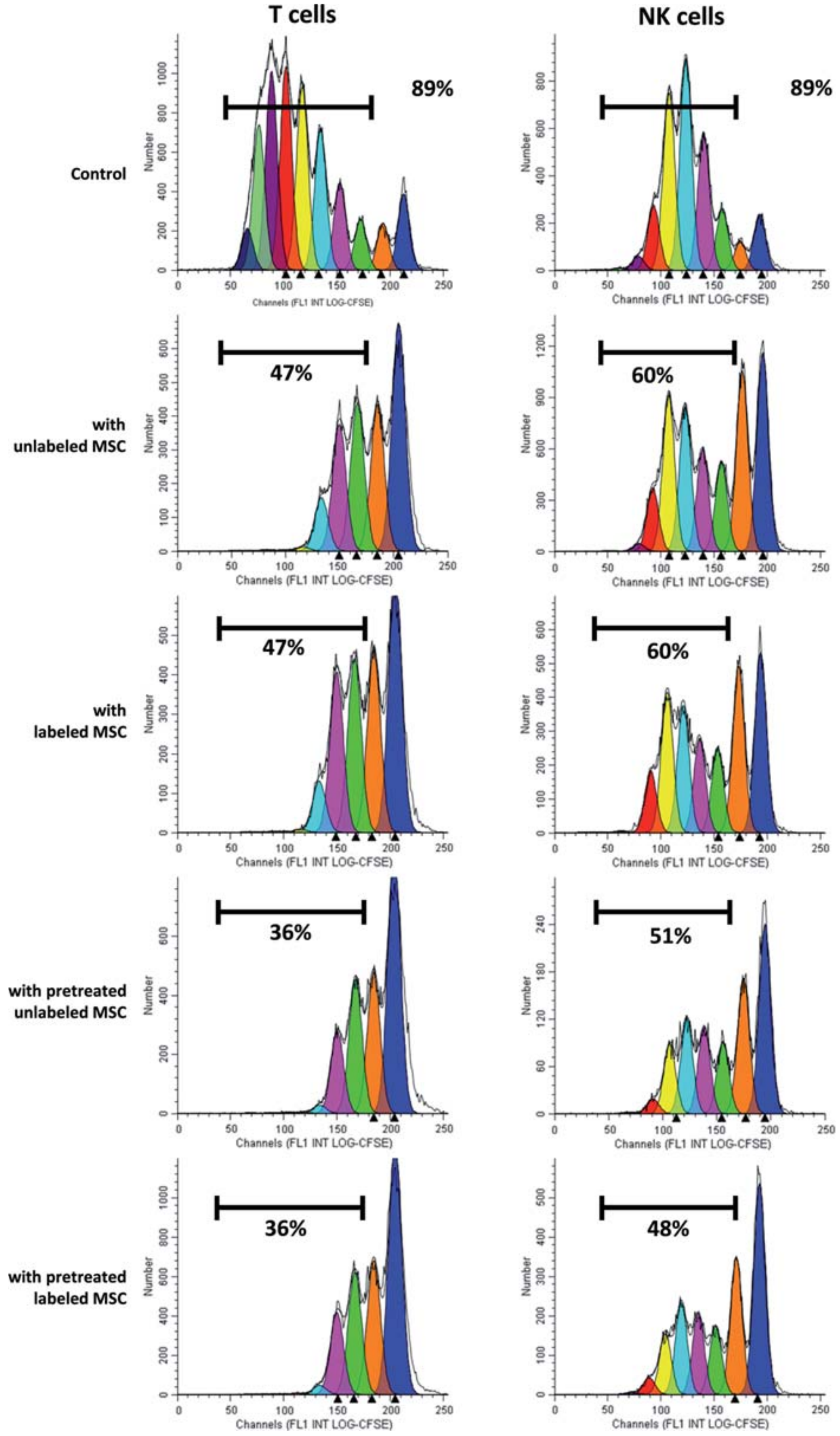

Supplementary Figure 7. Proliferation of purified T and NK cells in response to CD3/CD28 crosslinking or IL-2, respectively. CFSE dilution was evaluated on day 5 of culture without MSCs (first row panels), or in the presence of resting unlabeled (second row panels) or labeled (third row panels) MSCs, or in the presence of unlabeled (fourth row panel) or labeled MSC. MSCs (passage 1) were labeled with MU-Wuest4 (100 µg Fe/ml) for 24 h.((last row panel) pretreated for 2 days with IFN-γ/TNF-α before co-culture. Results are expressed as the percentage of T or NK cells that have undergone more than one cell division; one representative experiment of two.

Supplementary Table I. Relaxivity (r_2 and r_2*) of different iron oxide-PLLA particles in agarose 2% at 3 Tesla and room temperature.

Particle name	Iron oxide used for preparation	r_2 ($mM^{-1} \cdot s^{-1}$)	r_{2*} ($mM^{-1} \cdot s^{-1}$)
MU-Wuest1	wuestite (25 nm)	28.4	357.8
MU-Wuest2	wuestite (25 nm)	27.0	469.8
MU-Wuest3	wuestite (25 nm)	10.6	415.8
MU-Wuest4	wuestite (25 nm)	15.2	449.3
Resovist®	Carboxy-dextran coated iron-oxides	276.1	245.3

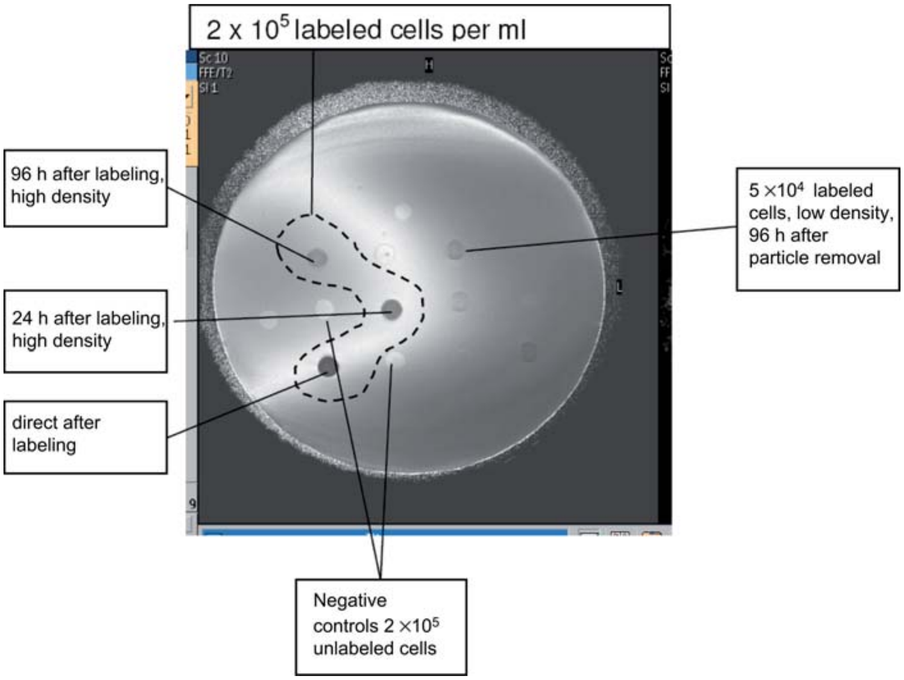

Supplementary Figure 8. Agarose phantom with either 2×10^4 or 3×10^3 MSCs/ml at 0, 24, 48 and 96 h after particle removal. MSCs (passage 7) were labeled with MU-Wuest3 100 µgFe/ml for 24 h. T_{2*} image with 0.25 mm resolution.
